# The impact of an unemployment insurance reform on incidence rates of hospitalisation due to alcohol-related disorders: a quasi-experimental study of heterogeneous effects across ethnic background, educational level, employment status, and sex in Sweden

**DOI:** 10.1186/s12889-022-14209-2

**Published:** 2022-10-03

**Authors:** Ylva B. Almquist, Alexander Miething

**Affiliations:** Department of Public Health Sciences, Centre for Health Equity Studies (CHESS), Stockholm University, 106 91 Stockholm, Sweden

**Keywords:** Unemployment insurance, Reform, Policy change, Health, Quasi-experimental, Registers, Sweden

## Abstract

**Background:**

Many Western countries have scaled back social and health expenditure, including decreases in the generosity and coverage of unemployment insurance, resulting in negative effects on general health and well-being at the aggregate level. Yet, research has not sufficiently looked into heterogeneity of such effects across different subgroups of the population. In Sweden, the 2006 unemployment insurance reform, implemented on the 1^st^ of January 2007, encompassed a drastic increase of insurance fund membership fees, reduced benefit levels, and stricter eligibility requirements. As this particularly affected already socioeconomically disadvantaged groups in society, such as foreign-born and low-educated individuals, the current study hypothesise that the reform would also have a greater impact on health outcomes in these groups.

**Methods:**

Based on register data for the total population, we utilise a quasi-experimental approach to investigate heterogeneous health effects of the reform across ethnic background, educational level, employment status, and sex. Due to behaviourally caused diseases having a relatively shorter lag time from exposure, hospitalisation due to alcohol-related disorders serves as the health outcome. A series of regression discontinuity models are used to analyse monthly incidence rates of hospitalisation due to alcohol-related disorders among individuals aged 30–60 during the study period (2001–2012), with the threshold set to the 1^st^ of January 2007.

**Results:**

The results suggest that, in general, there was no adverse effect of the reform on incidence rates of hospitalisation due to alcohol-related disorders. A significant increase is nonetheless detected among the unemployed, largely driven by Swedish-born individuals with Swedish-born or foreign-born parents, low-educated individuals, and men.

**Conclusions:**

We conclude that the Swedish 2006 unemployment insurance reform generally resulted in increasing incidence rates of hospitalisation due to alcohol-related disorders among unemployed population subgroups known to have higher levels of alcohol consumption.

**Supplementary Information:**

The online version contains supplementary material available at 10.1186/s12889-022-14209-2.

## Background

Previous research has shown that a generous and universal welfare state holds the capacity to protect its citizens against deleterious health effects of disadvantaged living conditions, thereby reducing the social gradient in health [1]. Yet, over the past decades, many Western countries have scaled back social and health expenditure, including decreases in the generosity and coverage of unemployment insurance [2]. Studies investigating such changes have demonstrated negative effects on general health and well-being at both the individual and the country-level [3, 4]. It is nevertheless likely that some segments of the population are more adversely influenced than other. Taking the 2006 unemployment insurance reform in Sweden as an example, the current study will utilise a quasi-experimental approach to investigate heterogeneous health effects across ethnic background, educational level, employment status, and sex.

Due to behaviourally caused diseases having a relatively shorter lag time from exposure, hospitalisation due to alcohol-related disorders will serve as the health outcome. In the Swedish context, the total alcohol consumption has been characterised by stability since the beginning of the 2000s [5] whereas a general reduction has been shown for risk consumption [6]. Concerning hospitalisation due to alcohol-related disorders, rates were slightly decreasing from the late 1990s until mid-2000s, after which a small increase could be noted. Between 2007 and 2012, hospitalisation rates were relatively stable before starting to decrease again [7]. Previous studies nonetheless suggest that more disadvantaged groups in society have a disproportionate level of consequences related to alcohol use [8]. Accordingly, we hypothesise that the 2006 unemployment reform led to increased incidence rates of hospitalisation due to alcohol-related disorders especially among these groups.

### The Swedish 2006 unemployment insurance reform

The Swedish welfare state has traditionally been characterised as generous [9]. Over time, however, policy changes and their accompanying reforms have resulted in a slow but persistent retrenchment [10]. A clear example of this can be found for unemployment insurance. For a long time, the Swedish system was based on a passive labour market policy with relatively generous and extended benefits without any strict qualifying requirements [11]. The voluntary unemployment insurance system was established in the 1930s and, while governed and subsidised by the state, it is managed by a relatively large set of unemployment insurance funds [12]. Important to note is that union membership automatically covers the fund membership fee. As of the late 1990s, the insurance system encompasses two schemes. First is the basic benefit scheme which is based on a daily allowance (*flat-rate benefits*). It is available to all job-seekers, including those who are not members of any fund, assuming that they meet certain requirements related to e.g. employment history. Second is the income-based scheme, which pays a replacement allowance (up to 80%) based on previous earnings but is conditioned upon membership in a fund (*earnings-related benefits*) [13].

Despite relatively low unemployment rates at the time, the public debate preceding the 2006 parliamentary election to a large extent circulated around the widespread perception of difficulties finding employment and discontentment with the idea of a high share of people receiving benefits [10, 14]. Making the reduction of unemployment one of their priorities, the alliance of center-right parties that won the election immediately introduced a substantial reform of the unemployment insurance which came into effect January 1, 2007. While the basic setup of the system remained largely the same, the state considerably decreased its involvement by reducing contributions to the unemployment insurance funds. As a consequence, union membership fees increased and the amount of benefits was reduced, which ultimately led to a significant drop in members of unemployment insurance funds. Although Sweden still has one of the highest unionisation rates in the world, union density fell by four percentage points between 2006 and 2007 (from an annual average of 77% to 73% among individuals aged 16-64) [15]. This was not only caused by individuals deciding to leave the unemployment insurance funds but also by a decreasing inflow of new members. Past research has shown that the drop was particularly evident among funds targeting the working class, for which the membership fees also increased more drastically [16]. Such funds generally have a higher representation of individuals with lower educational attainment and occupations generating less income, as well as foreign-born. Indeed, studies show that those who had resided in Sweden for a short period of time and originated from countries that are geographically far from Sweden were considerably more likely to drop out of the unemployment insurance funds [17]. Additionally, incentives for accessing the income-based scheme are most likely weaker among individuals with low income [18]. Concerning differences by sex, the decrease in members varied greatly across specific funds without any systematic differences between male-dominated and female-dominated funds [16].

The aforementioned changes of the unemployment insurance also involved requirements for benefits being made stricter [15]. This led to a decrease in the share of unemployed receiving unemployment insurance benefits, whether accessed through the basic benefit scheme or income-based scheme. For example, comparing the periods 2001-2006 and 2007-2012, the share of unemployed receiving such benefits diminished by approximately one half [19]. These individuals were instead referred to other sources of financial support, such as social assistance benefits [20]. Again, groups that were particularly affected included individuals with weak labour market attachment, such as foreign-born [19].

### Heterogeneous effects of the 2006 unemployment insurance reform

Previous research has identified several plausible pathways linking unemployment insurance to health-related outcomes [3]. By definition, unemployment benefits compensate (at least partially) for income loss among the unemployed. In addition to securing the economic resources required to satisfy the individual’s basic needs, it allows for investments in goods and activities that can further promote health [21, 22]. However, an unemployment insurance system that is generous in terms of benefit levels and coverage may be regarded as a collective resource [23] that is beneficial also for the employed. While unemployment insurance in itself cannot directly decrease the risk of becoming unemployed, the perception of economic security (in case of job loss) it entails could reduce feelings of stress and anxiety and thereby positively affect health [22]. In particular, such spill-over effects are expected to apply to groups who have employment but still experience limited economic resources and weaker labour market attachment [23].

More specifically applied to alcohol-related disorders, previous studies have found unemployment to be a risk factor [24]. Unemployment – or the threat of unemployment – may also worsen already existing substance use problems and increase the risk of relapse [25]. A reduction in the generosity and coverage of unemployment insurance could potentially exaggerate stress related to the anticipation of job loss or experiences of unemployment, and thereby increase the use of alcohol as a way to cope with stress, which would then magnify the risk of hospitalisation due to alcohol-related disorders [cf. 3, 17]. In this context, having a low educational level – and therefore potentially less socioeconomic resources – might reduce the individuals’ overall ability to manage their experiences of unemployment and exacerbate the negative impact on (alcohol-related) behaviours. Moreover, drawing upon previous studies looking into sex differences in the effects of unemployment on health-related outcomes [26], any such processes are likely to be more evident among men.

When it comes to individuals with foreign background living in Sweden, previous studies have generally demonstrated lower rates of alcohol consumption and alcohol-related disorders as compared to natives [27]. Individuals with foreign background is nonetheless a highly diverse group, and there are some marked differences between first-generation and second-generation immigrants as well as according to country of origin [28]. For example, rates tend to converge by time spent in Sweden and be relatively higher among groups originating from countries with an alcohol culture more similar to Sweden [27]. Regardless of the lower rates, however, there is reason to believe that stressful situations – such as facing unemployment – can be more detrimental for a population that experience more disadvantaged living conditions on average, and weaker labour market attachment in particular [29, 30]. Combining this with the observation that unemployment insurance coverage and replacement rates among foreign-born individuals were greatly affected by the 2006 unemployment insurance reform, it is plausible that influences of the reform on alcohol-related disorders might also be more pronounced in this group.

### Aim and research questions

The overall aim of this study is to study (heterogeneity in) the effects of the 2006 unemployment insurance reform on hospitalisation due to alcohol-use disorders, using a quasi-experimental approach. Our study period is 2001–2012, with January 1, 2007 as the threshold of interest. The specific research questions are as follow:

- 1A. How did incidence rates of hospitalisation due to alcohol-related disorders respond to the unemployment insurance reform?
- 1B. To what extent does any such effect look different across subgroups of the population, as defined by ethnic background, educational level, employment status, and sex?

In order to expand our understanding one step further, we will specifically examine heterogeneous effects among the unemployed individuals and the role played by unemployment benefit recipiency. Thus, here we ask:

- 2A. To what extent does any effect of the reform look different across subgroups of the unemployed population, as defined by ethnic background, educational level, and sex?
- 2B. Does recipiency of unemployment benefits play a role for any observed heterogeneity in effects?

## Methods

This repeated cross-sectional study employs Swedish longitudinal population and medical registries that cover the total registered population residing in Sweden from January 2001 to December 2012. Pseudonymised unique identification numbers were used to link individuals’ socioeconomic and demographic characteristics obtained from Statistics Sweden with medical data from the Swedish National Board of Health and Welfare. Ethical approval of this study was granted by the Swedish Ethical Review Authority (decision no. 2021–06797-01).

### Study sample

The initial sample included individuals in ages 30 to 60 years at any point during the study period (*n* = 5,759,292). We right-censored individuals who died or migrated during or before the study period (*n* = 637,860) and excluded those with missing information on ethnic background or education (*n* = 103,159). Additionally, we excluded those for whom employment status could not be ascertained (*n* = 8,441). These exclusions led to 5,009,832 individuals being included in the study population (see Additional file 1, Supplementary Figure S1, for an overview). The unit of analysis in the current study is however not individuals but monthly aggregates of the individual-level data.

### Study variables

The stratification variables comprised ethnic background, educational level, employment status, and sex. In addition, among the unemployed, we included information about unemployment benefit recipiency. Hospitalisation due to alcohol-related disorders served as study outcome.

#### Ethnic background

Information on ethnic background was derived from Statistics Sweden’s longitudinal database for integration studies (STATIV) and the multigenerational database. Based on nativity and region of origin, we distinguished between four groups in this study: (i) Swedish-born individuals with Swedish-born parents, (ii) Swedish-born with at least one foreign-born parent, and (iii) European Foreign-born, and (iv) non-European foreign-born.

#### Educational level

A dichotomous variable was used to account for the individuals’ highest attained level of education during the follow-up period. Individuals with primary and secondary education were categorised as low-educated. The high-educated category comprised individuals with tertiary education, i.e., those who completed longer vocational trainings and received academic degrees.

#### Employment status

Measures from Statistics Sweden’s Longitudinal Integration Database for Health Insurance and Labour Market Studies (LISA) were used to distinguish between (1) individuals in employment and self-employment, and (2) individuals who experienced episodes of unemployment. The use of longitudinal data with multiple observations for each individual implicates that a substantial share of individuals experienced (3) episodes of both employment as well as unemployment. In order to properly distinguish between ‘being employed’ and ‘being unemployed’, we stratified the study population on the basis of unique observations instead of individuals. Whereas the first strata included observations of individuals being employed or self-employed, the second subset comprised all records of unemployment. Despite this clear-cut distinction, the observations in either category might refer to one and the same individual due to changes in employment status over the study period. It should be noted that information on employment and records of unemployment obtained from LISA is only provided on annual basis and refers to the individuals’ status in November of each year. Because this study employs month-by-month cross-sections as the unit of analysis, the annual records of employment status were linked to the monthly aggregates of hospitalisations due to alcohol use. This was done by expanding the annual employment information from wide into long data format and merging it with the hospitalisation data.

#### Sex

Individuals were categorised as either “men” or “women”.

#### Unemployment benefit recipiency

Unemployed individuals were in addition categorised according to whether they received (flat-rate benefits or earning-related) benefits or not.

#### Hospitalisation due to alcohol-related disorders

Information on hospitalisation due to alcohol-related disorders was drawn from the inpatient care registers maintained by the Swedish National Board of Health and Welfare. All inpatient care events with overnight stays in Swedish hospitals linked to the diagnosis F10 (‘Mental and behavioural disorders due to alcohol use’) in Chapter V of the International Classification of Diseases (ICD-10) during the period 2001 to 2012 were included in the analysis.

For each calendar month within the study period (2001–2012), and for each level of the stratifying variables, we calculated the incidence rate of hospitalisation due to alcohol-related disorders (number of hospitalisations per 1,000 person-years).

### Statistical analysis

The analysis uses a quasi-experimental study approach based on a regression discontinuity design (RDD) [31]. RDD is well-established in economics and has become increasingly common in other research disciplines, including epidemiology. The basic idea of RDD is to assess the effects of an intervention (usually referred to as ‘treatment’) by assigning a threshold between the treatment and control group. Observations on either side of the threshold are compared and the average treatment effect is then measured with the emerging discrepancy (usually referred to as ‘discontinuity’) between these treatment and control units. RDD requires relatively mild assumptions compared to other nonexperimental research designs [32]: the basic model involves a continuous scoring variable representing the outcome, a forcing variable representing the exposure, and an (exogeneous) treatment to determine the cut-off (or ‘threshold’) when the discontinuity occurs. Furthermore, a key feature of RDD is the regression discontinuity (RD) plot. It is obtained by plotting the local means of incidence rates over units of time and estimating distinct regression lines for the treatment and control units. The gap between the intercepts of both regression lines around the cut-off-point designates the magnitude of regression discontinuity and the quasi-causal effect of the intervention of interest. The current study employs ‘Regression discontinuity in time’ (RDiT). RDiT is considered a subtype of RDD and involves units of time as the forcing variable [33]. The study period was 2001–2012 and the cut-off (threshold) was set at the date of the intervention (January 1^st^ 2007). All analyses were performed with Stata v17.

Based on the Stata package RDROBUST, a default RD model with uniform kernel function and evenly spaced mimicking-variance (ESMR) was used. The estimated regressions were based on local quartic (4^th^ order) polynomials that were derived from non-parametric regressions [34]. In the first set of models, we included both employed and unemployed individuals. The analysis was performed for the total study population and then separately by ethnic background, educational level, employment status, and sex. In the second set of models, the analysis was first performed for the unemployed population and then separately by ethnic background, educational level, and sex.

A corresponding RD plot was generated for each model to illustrate the results, with help of the user-written Stata packages RDPLOT [35] and HEATPLOT [36]. The RD plots are based on the local means of group-specific incidence rates of hospitalisation due to alcohol-related disorders at each month during the study period (represented by bins in the plots). The vertical dashed line denotes the cut-off point (January 1, 2007) that divides the time axis into a pre-intervention period and a post-intervention period. In the RD plots for the unemployed population, coloured bins were used to depict the altering proportions of between receiving benefits versus not receiving benefits at each cross-section; a higher value (and hence a warmer colour) indicates a larger proportion of non-recipients. Model-based regression lines were included in all RD plots to demonstrate the change around the threshold. Moreover, confidence intervals are included as shaded areas in the RD plots.

#### Robustness checks

In order to validate the findings, we performed several robustness checks based on the second set of models (i.e. for the unemployed population only). To start with, we contrasted the regression discontinuity occurring in January 2007 with arbitrarily chosen cut-off points. We ran models that examined changes in incidences of alcohol-related disorders around January 2004 and January 2010; time points that are timely remote from the studied unemployment insurance reform. As we were primarily interested in comparing the altering intercepts, regressions based on linear (1^st^ order) polynomials were applied.

Furthermore, the study period that we choose in this study is relatively long (2001–2012). In order to obtain more robust effects of the reform as well as to circumvent problems with competing reforms occurring during the post-intervention period, we ran models with a more conservative bandwidth (2005–2008). Regressions were based on quartic and linear polynomials, respectively, to additionally explore robustness in relation to the choice of function. Confidence intervals were included as shaded areas.

## Results

### Descriptives

The descriptive characteristics of the study population are shown in Table 1. The estimates demonstrate higher incidence rates of hospitalisation due to alcohol-related disorders among men in relation to women, comparably high numbers in the European foreign-born population, but low rates among the Non-European foreign-born. Large differences in incidence rates by educational level and employment status are shown. Among individuals who were unemployed but did not receive unemployment benefits, incidence rates are more than twice as high compared to those unemployed who received such benefits. Unemployed and individuals with low education also show a notable increase across the two specified periods (2001–2006 and 2007–2012).

**Table 1:** Descriptive characteristics of the study population, 2001–2012

			**Hospitalisations due to alcohol-related disorders**				
	**Number of individuals**	**Observations (person-years)**	**Number of** **hospitalisations**	**Number of hospitalisations** **per 1,000 person-years**			
				**2001–2006**	**2007–2012**	**2006**	**2007**
**Study population**	5,009,832	42,370,094	189,874	4.4	4.5	4.3	4.5
**Ethnic background**							
Swedish-born with Swedish-born parents	3,772,685	32,678,931	148,611	4.4	4.7	4.3	4.5
Swedish-born with foreign-born parent(s)	322,466	2,905,889	14,022	4.3	5.3	4.4	5.0
European foreign-born	470,518	3,572,271	23,468	7.1	6.0	6.9	6.6
Non-European foreign-born	444,163	3,213,003	3,773	1.1	1.2	1.2	1.2
**Education**							
Low education	3,138,150	27,209,211	167,239	5.9	6.4	5.9	6.2
High education	1,871,682	15,160,883	22,635	1.5	1.5	1.4	1.4
**Employment status**							
Employed	3,563,123	38,164,980	148,435	4.0	3.8	3.8	3.9
Unemployed	1,446,709	4,205,114	41,439	8.2	11.7	8.6	10.7
Unemployment benefit recipiency	1,015,190^a^	2,668,090	17,839	5.9	7.9	6.2	7.8
Unemployment benefit non-recipiency	836,098^a^	1,537,024	23,600	14.0	16.3	13.7	15.4
**Sex**							
Men	2,547,349	21,658,406	145,427	6.7	6.7	6.5	6.7
Women	2,462,483	20,711,688	44,447	2.0	2.3	2.0	2.1
	**Mean**	**Standard deviation**					
**Age**	45.0	8.9					

^a^Because unemployment benefit (non-)recipiency varies over time, the total number of individuals in this variable exceeds the number of individuals in the study population

Additional files 2, 3 and 4 show the percentage of unemployed individuals with and without unemployment benefit recipiency by ethnic background, educational level, and sex. It can be noted that the percentage of unemployed individuals was generally stable or slightly declining in most groups before the reform was implemented. A more evident decrease is then seen in 2007–2008, followed by an increase starting in 2009. Concerning the results stratified by ethnic background (Additional file 2; Supplementary Figure S2), the orange lines disclose that the share of unemployed Swedish-born with Swedish-born parents who received benefits declined between the years 2005 and 2009 (two years before and after the reform was implemented). This pattern is consistent throughout all subgroups. The share of unemployed individuals without unemployment benefit recipiency is largely stable for both groups of Swedish-born, with some tendency towards an increase over time. For European foreign-born and non-European foreign-born, there is a marked increase starting around 2008, especially in the latter group. The blue bars depict how recipiency versus non-recipiency deviate from the overall mean among the unemployed. Particularly obvious is the mirror-inverted timely pattern with a breaking point in the year 2007 in the group of non-European foreign-born. The plots that stratify by education (Additional file 3; Supplementary Figure S3) and sex (Additional file 4; Supplementary Figure S4) show largely similar patterns. Among the low-educated, the increase of non-recipients after 2007 is somewhat more pronounced compared to the high-educated.

### Results from the regression discontinuity analyses

**Fig. 1:**
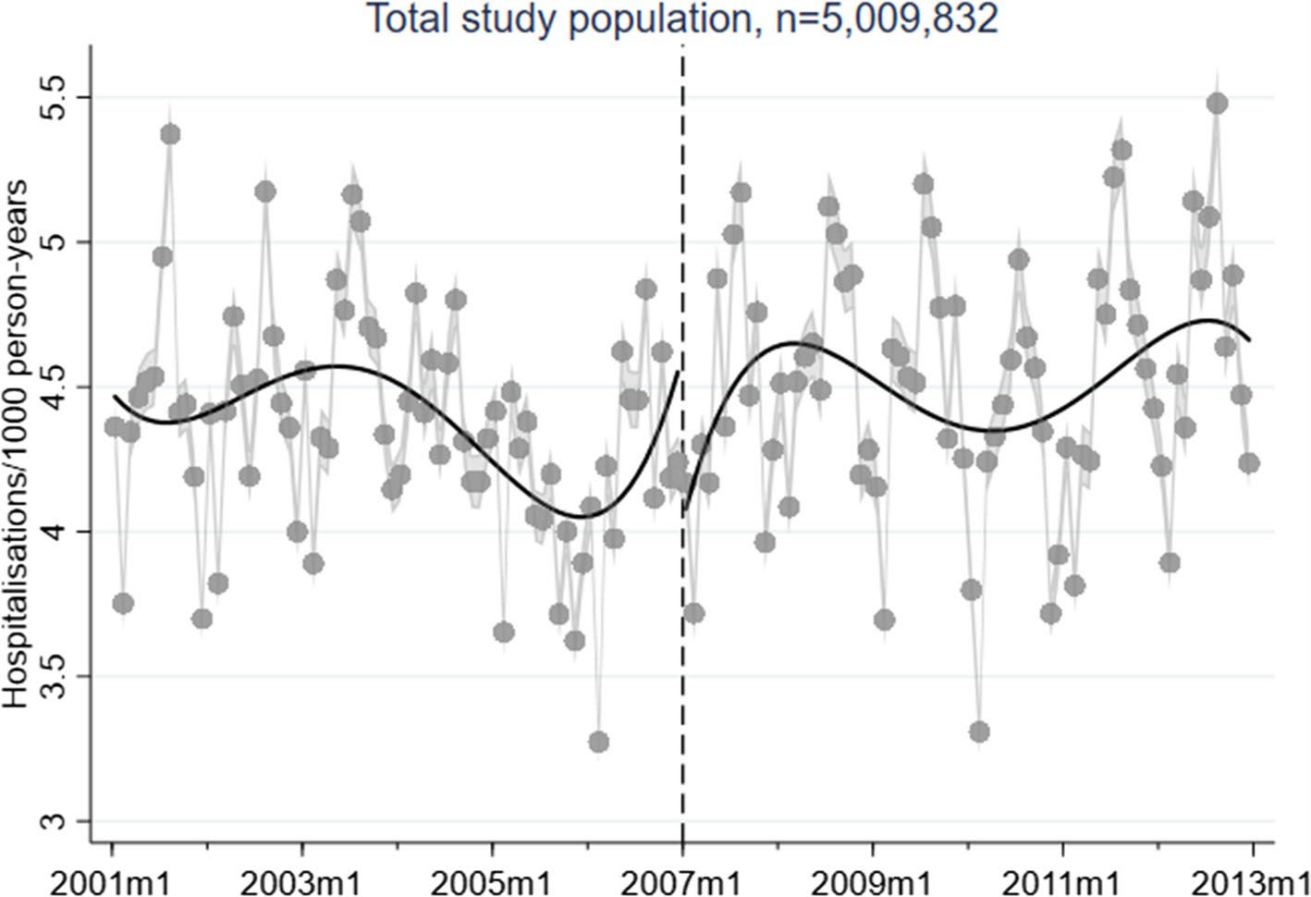
Regression discontinuity plots with incidence rates of alcohol-related disorders. Total study population (ages 30–60, 2001–2012)

**Fig. 2:**
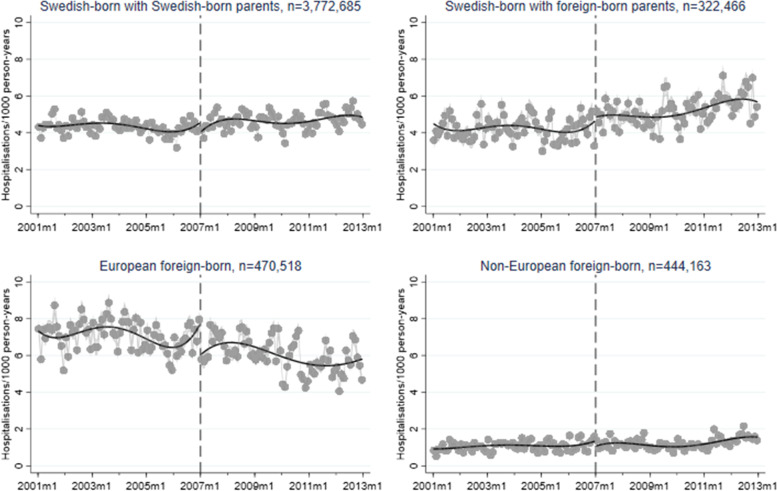
Regression discontinuity plots with incidence rates of alcohol-related disorders. Total study population, stratified by ethnic background (ages 30–60, 2001–2012)

**Fig. 3:**
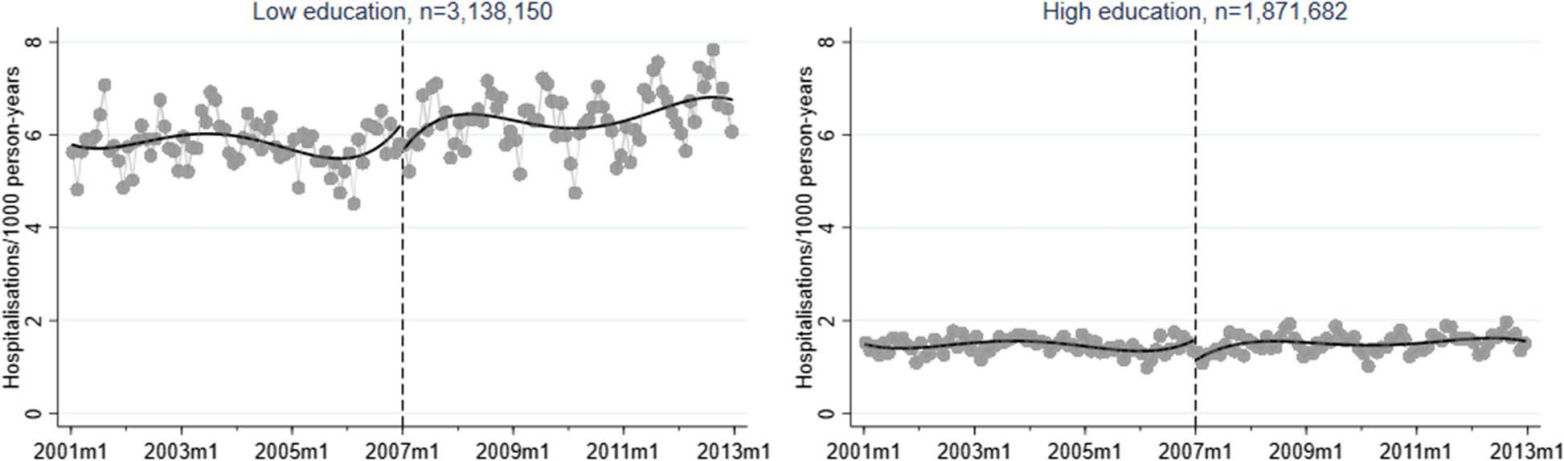
Regression discontinuity plots with incidence rates of alcohol-related disorders. Total study population, stratified by educational level (ages 30–60, 2001–2012)

**Fig. 4:**
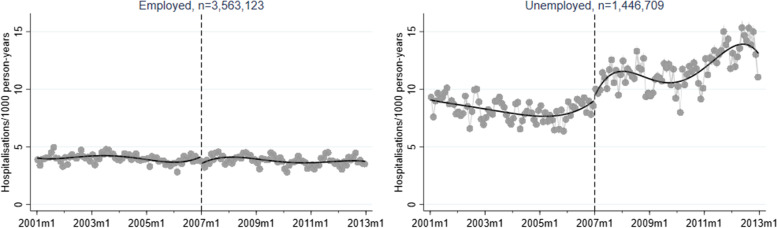
Regression discontinuity plots with incidence rates of alcohol-related disorders. Total study population, stratified by employment status (ages 30–60, 2001–2012)

**Fig. 5:**
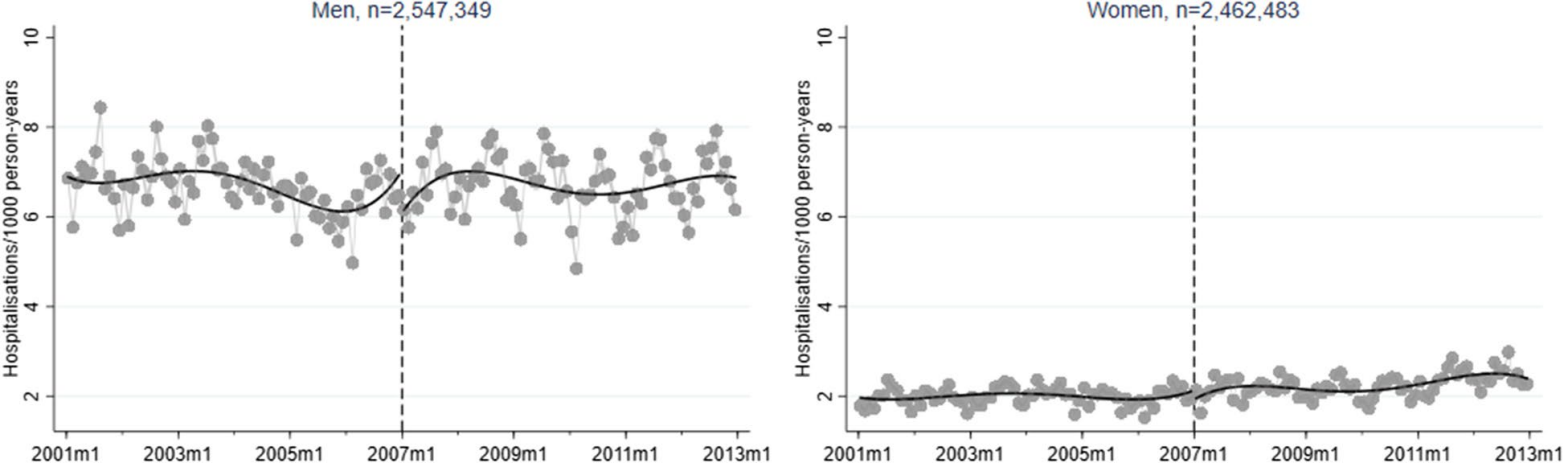
Regression discontinuity plots with incidence rates of alcohol-related disorders. Total study population, stratified by sex (ages 30–60, 2001–2012)

Figures 1, 2, 3, 4, and 5 show RD plots with incidence rates of hospitalisation due to alcohol-related disorders during the period 2001–2012 in the total study population, as well as by ethnic background, educational level, employment status, and sex. Again, it should be noted that the bins represent the local means of group-specific incidence rates of hospitalisation due to alcohol-related disorders at each month during the study period. The point estimates, *p*-values, and confidence intervals are available in Additional file 5 (Supplementary Table S1).

Regarding the results for the total study population (Fig. 1), the results show a slight reduction in incidence rates around the time that the reform was implemented (-0.49, *p* < 0.001), which is expected based on the overall decrease of hospitalisation due to alcohol-related disorders over time.

Next, the results are stratified by ethnic background (Fig. 2). Swedish-born with Swedish-born parents show a small decrease in incidence rates (-0.42, *p* < 0.001). This is also the case among non-European foreign-born individuals (-0.62, *p* < 0.001) although their absolute levels of hospitalisation are consistently much lower in comparison to the other groups. A more pronounced decrease can be seen for European-foreign individuals (-2.39, *p* < 0.001). In contrast, increased incidence rates posterior to the intervention are found among Swedish-born with foreign-born parents (1.41, *p* < 0.001).

Stratification by educational level (Fig. 3) discloses substantial differences in absolute levels between individuals with low and high education. However, both groups demonstrate a small decrease around the time of the reform (low education: -0.47, *p* < 0.001; high education: -0.43, *p* < 0.001).

For the results stratified by employment status (Fig. 4), the slight decrease in incidence rates for employed individuals (-0.56, *p* < 0.001) is contrasted by a relatively sharp increase among unemployed around the year 2007 (1.04, *p* < 0.001).

Large absolute differences in incidence rates can be observed between men and women (Fig. 5). The comparably high incidence rates among men follow the pattern seen for the total population. For both groups, however, decreases in the incidence rate of hospitalisation due to alcohol-related disorders can be observed (men: -0.77, *p* < 0.001; women: -0.21, *p* < 0.001).

**Fig. 6:**
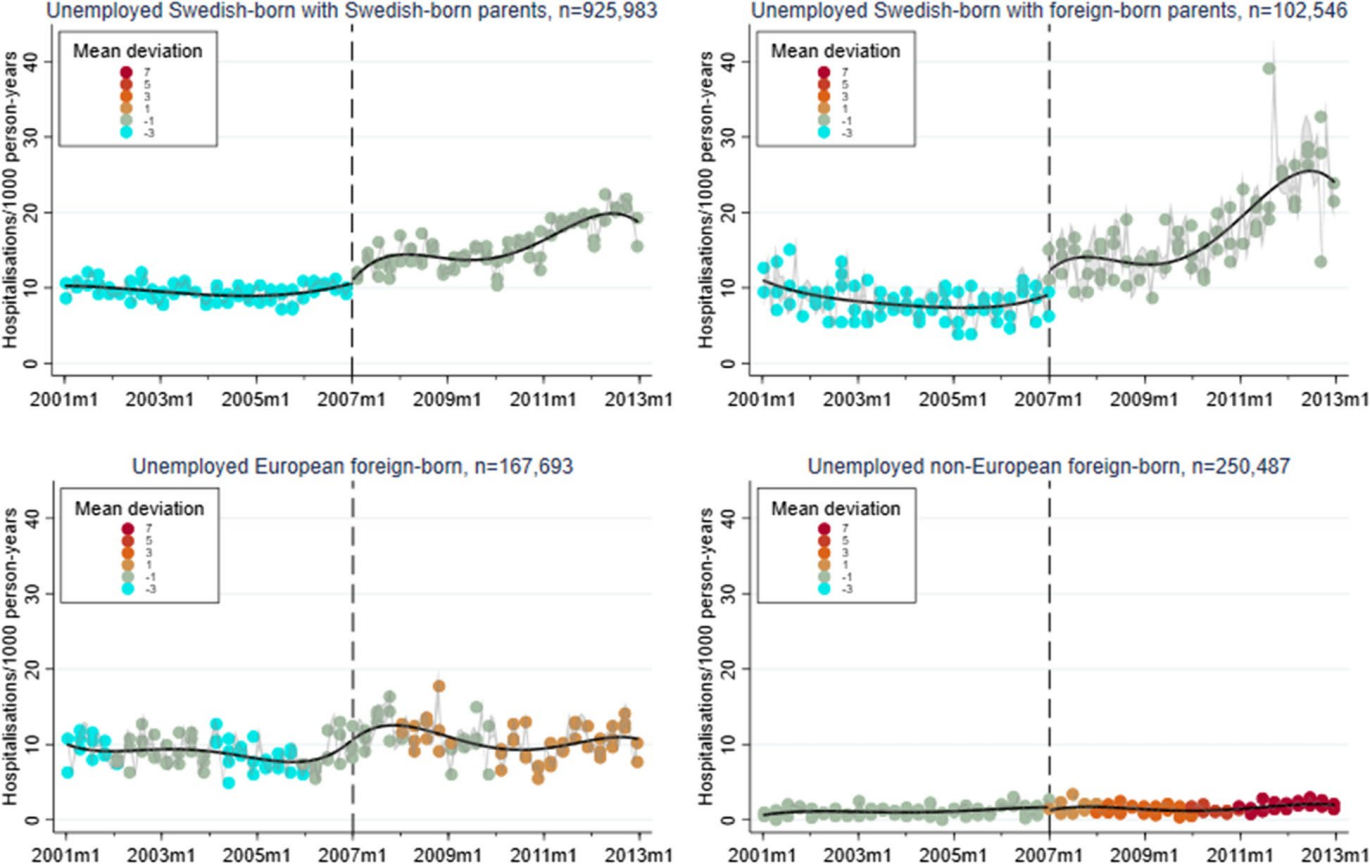
Regression discontinuity plots with incidence rates of alcohol-related disorders. Unemployed population, stratified by ethnic background (ages 30–60, 2001–2012)

**Fig. 7:**
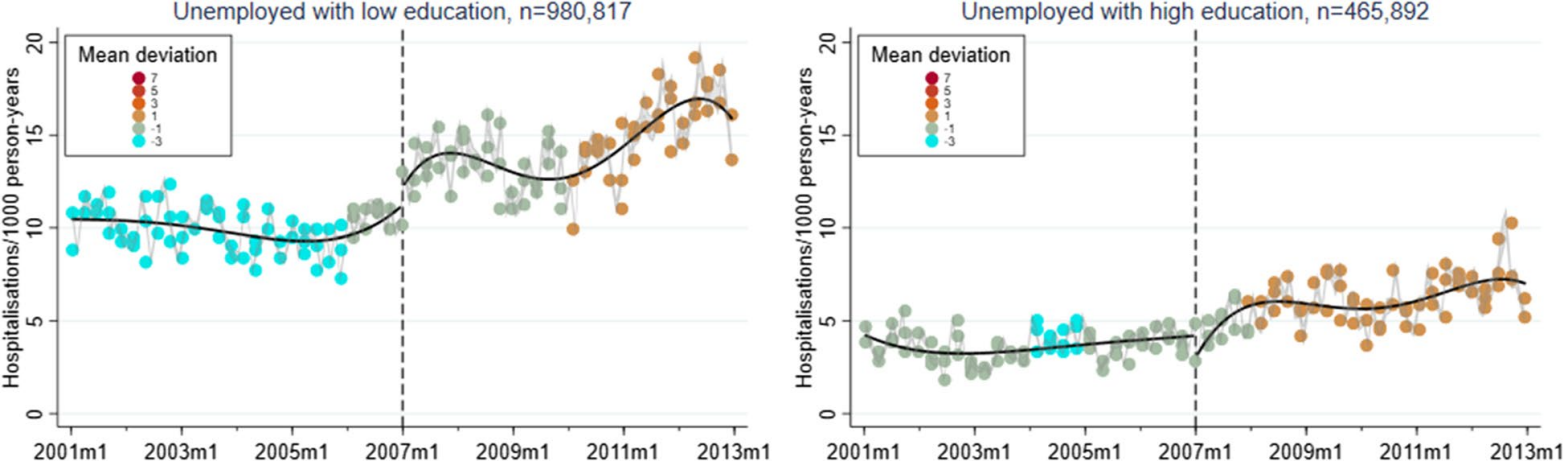
Regression discontinuity plots with incidence rates of alcohol-related disorders. Unemployed population, stratified by educational level (ages 30–60, 2001–2012)

**Fig. 8:**
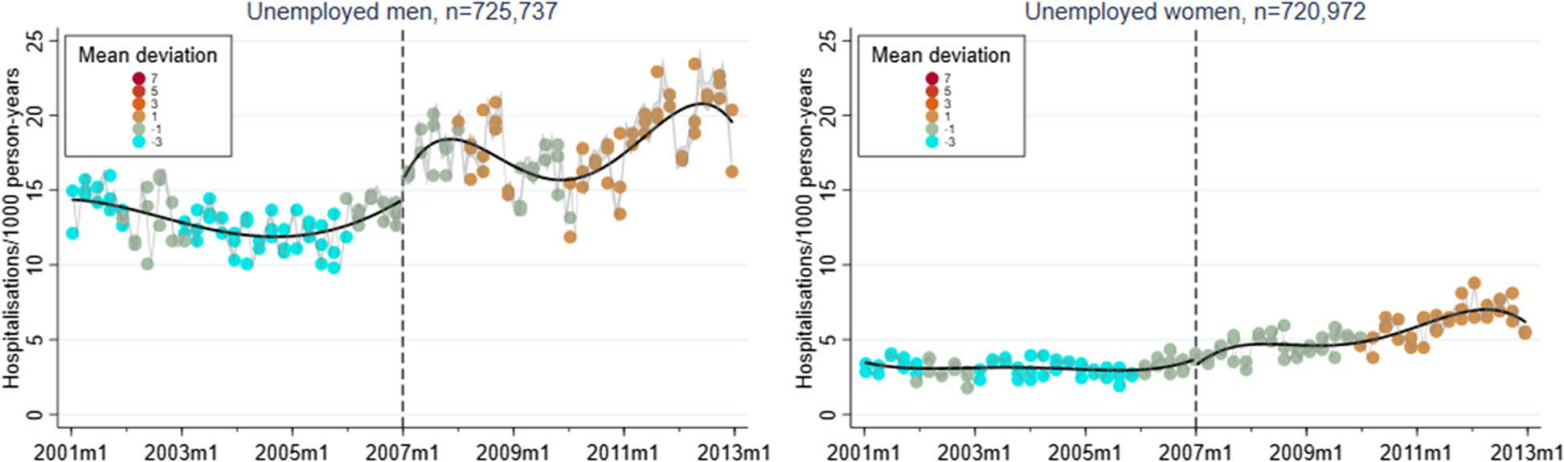
Regression discontinuity plots with incidence rates of alcohol-related disorders. Unemployed population, stratified by sex (ages 30–60, 2001–2012)

The next set of figures (Figs. 6, 7 and 8) show the results specifically for the unemployed population, stratified by ethnic background, educational level, and sex. As previously mentioned, coloured bins were used to illustrate changes in the absolute difference between unemployed who received unemployment benefits and unemployed who did not receive such benefits during the study period.

Focusing on unemployed individuals’ ethnic background (Fig. 6), Swedish-born with Swedish-born parents show upward trends after January 2007 when the unemployment insurance reform was implemented (2.25, *p* < 0.001). An increase can also be noted for European foreign-born individuals (3.83, *p* < 0.001). Among the Swedish-born with foreign-born parents, the effect of the reform is particularly evident (7.41, *p* < 0.001). On the contrary, Non-European foreign-born individuals show very low incidence rates with a marked decrease around the time of the reform (-2.05, *p* < 0.001). Interesting to note is that the increase in the proportion of non-recipients during the post-intervention period seems to be largest for the non-European foreign-born individuals and smallest for the Swedish-born with foreign-born parents.

In terms of educational background (Fig. 7), incidence rates are comparatively higher among low-educated individuals. For this group, there is also a notable increase around the time of the reform (2.50, *p* < 0.001). The opposite is demonstrated for high-educated individuals (-1.52, *p* < 0.001). Men disclose incidence rates that are twice as high compared to women (Fig. 8). Both sexes demonstrate an increase in incidence rates due to alcohol-related disorders, although this is more pronounced among men (men: 2.77, *p* < 0.001; women: 0.51, *p* < 0.001). In general, a gradual increase in the proportion of non-recipients can be noted in all groups.

#### Robustness checks

The first series of sensitivity analysis (see Additional files 6, 7 and 8; Supplementary Figures S5-S7) examines the robustness of the selected threshold. In general, the alternative cut points January 2004 and January 2010 do not show larger increments in alcohol-related disorders compared to January 2007 when the unemployment insurance reform was implemented. This is particularly clear among some of the groups within the unemployed population that previously showed the largest effects, including Swedish-born with Swedish-born parents, European foreign-born individuals, low-educated individuals, and men. Considering that the differences between the cut points are less pronounced among Swedish-born with foreign-born parents and women, the previously identified effects in these groups should be interpreted with caution.

The next series of robustness checks (see Additional files 9, 10 and 11, Supplementary Figures S8-S10) examine the effect of the reform using a narrower bandwidth (2005–2008). Regardless of whether the regression lines were based on quartic or linear polynomials, the results largely mirror the analyses using the entire follow-up period (2001–2012).

## Discussion

The current study set out to explore how incidence rates of hospitalisation due to alcohol-related disorders responded to the Swedish 2006 unemployment insurance reform. We were not able to identify any adverse effect of the reform in the total study population of individuals who were aged 30–60 during the period 2001–2012. Rather, the results followed the general trend during this period with only slight changes in the rates of hospitalisation due to alcohol-related disorders. This was largely also the case when stratifying by ethnic background, educational level, and sex.

Differentiating the results by employment status did, however, show a different picture. Others have previously argued that the threat of unemployment, especially in light of reforms that decrease the generosity of the welfare state, could induce feelings of stress and anxiety also among those who are gainfully employed [22]. We were not able to confirm such spill-over effects. There might be several explanations to this discrepancy, including the choice to focus on hospitalisation due to alcohol-related disorders as the outcome. Alcohol use is only one of many ways of coping with stress; it could be the case that employed individuals to a greater extent practice coping strategies that do not involve alcohol use or other potentially health-damaging behaviours [37]. Moreover, events that require inpatient care reflect a high degree of severity: we might have reached other conclusions if a different (and more general) outcome had been examined.

For the unemployed individuals, however, there was a clear indication of increasing incidence rates around the time when the 2006 unemployment insurance reform was implemented. Considering that hospitalisation due to alcohol-related disorders in these ages to larger extent reflects chronic disease (e.g. dependence) rather than acute events (e.g. intoxication), it is likely it has been preceded by a history of excessive alcohol use or problematic relationship to alcohol. In this context, the reform could have acted as a catalyst among individuals who were already in a vulnerable position to increase or relapse to health-damaging behaviours [38, 39]. This further points to the relevance of considering the contribution of health selection, especially bearing in mind that unemployment rates generally were declining in the years around the studied reform. Previous studies have shown health selection to be more evident in times when unemployment rates are low [40]. It is thus plausible that the composition of the unemployed population changed in important ways over the course of our follow-up. While it was outside the scope of the current study to empirically disentangle this issue, it remains an important task for future research.

To further explore heterogeneity specifically in the unemployed population, the analyses were stratified by ethnic background, educational level, and sex. The findings suggested that there was, in particular, a significant increase in incidence rates of hospitalisation due to alcohol-related disorders among unemployed individuals who were Swedish-born (regardless of whether they had Swedish-born or foreign-born parents), had low educational level, or were men. In terms of ethnic background, we initially hypothesised that the effect might be more pronounced among foreign-born individuals due to their overall weaker labour market attachment and, in particular, higher drop-out rate from the unemployment insurance funds [16, 17]. This initially appeared to be the case for European foreign-born individuals whereas the opposite was found for the non-European foreign-born. However, it should be noted that analysis of alternative cut points did not confirm the presence of clear effects due to the reform in any of these groups. Rather, it seems to be the case that subgroups that are known to have higher levels of alcohol use [41–43] were more vulnerable to the 2006 unemployment insurance reform. The results for educational level and sex lend further support to this notion.

The regression discontinuity plots also incorporated information about recipiency versus non-recipiency of unemployment benefits. Similar to what has been reported previously [19] there were striking increases over the study period with regards to the share of non-recipients. Yet, the subgroups experiencing the largest increase of non-recipiency were not the same as those showing the strongest indication of regression discontinuity – this was especially the case for non-European foreign-born individuals for whom the proportion drastically increased in the post-intervention period. Moreover, the increases were similar across educational levels and sexes. Since the consequences of the reform were hypothesised to be mediated through deteriorating financial security, the results might be taken as counterevidence for any causal effect of the 2006 unemployment insurance reform. However, even if the change in the proportion of unemployed individuals who did not receive unemployment benefits looks largely the same across most of the subgroups, it is still highly plausible that this had more severe consequences among those who already had relatively higher levels of alcohol consumption.

### Strengths and limitations

A major strength of the current study was the use of a total population sample which rendered sufficient statistical power to investigate a relatively rare outcome such as hospitalisation due to alcohol-related disorders while still being able to stratify the analysis into several subgroups. Another strength was that the study was based on register data with nearly complete coverage and limited loss to follow-up. The register-based exposure and study outcome was insusceptible to self-reporting bias or other sources of manipulation, which strengthens the validity of the RD design [32]. Moreover, even though the study was based on observational data, we could approach the issue of causality with more certainty by applying a quasi-experimental design.

Some important limitations should nevertheless be addressed. With regards to the outcome, hospitalisation data was the only feasible alternative since other national health registers in Sweden (e.g. the Prescribed Drug Register, and outpatient care data from the National Patient Register) did not commence until later years. As previously mentioned, this means that our outcome only captures the most adverse consequences related to alcohol use. Having information also on alcohol problems treated in outpatient care or alcohol consumption data might have provided a more complete picture of the effects of the 2006 unemployment insurance reform on alcohol-related outcomes.

The fact that the studied reform was implemented at the national level on a specific date (January 1^st^ 2007) is strength. However, it is unlikely that the reform caused any effects on incidence rates of hospitalisation due to alcohol-related disorders on the exact day of implementation. As with many reforms, the threshold is in reality ‘fuzzy’ – involving both anticipatory effects and lagged effects. We tried to account for this by truncating the regression lines three months before and after threshold in a series of sensitivity analyses (data not presented), but this did not cause the main conclusions of the study did not change. There are also other limitations with the regression discontinuity design that need to be discussed. To facilitate the interpretation, we included rather broad categories of ethnic background, educational level, employment status, and sex. For example, with regards to ethnic background, exploring differences across countries of origin is something for future research to consider, as it may provide a deeper understanding of the role of drinking cultures and level of alcohol use. Specifically, in this context, it may also be relevant to broaden the outcome to include other types of substance use.

There is always a possibility that effects found with regression discontinuity design are statistical artefacts, created by natural variation in the studied outcome over time, or that they are actually caused by macro-level changes other than the one targeted by the study [33]. To account for the former, we performed additional analyses with arbitrarily chosen cut-off points [44]. None of those showed any substantially different results in relation to the most prominent effects found in the main analyses, which strengthened our conclusions. In terms of the latter, it is indeed difficult to isolate the effect of a specific reform, since societies are in constant flux. We have not identified any similarly comprehensive reforms of relevance occurring at the same time as our specified threshold, neither in relation to unemployment insurance, alcohol policy, or health care. It should nonetheless be mentioned that several drastic macro-level changes occurred during the post-intervention period, including the financial crisis in 2008. Even if there were early global signs of a great recession already in 2007, the consequences did not hit the Swedish economy and labour market until late 2008. Compared to countries like, for example, Spain and Iceland, impacts on the Swedish economy were relatively mild [45, 46]. However, in order to decrease the possible influence of the financial crisis on our results, we re-examined our models using a narrower bandwidth (2005–2008). This did not cause our conclusions to change in any substantial way.

## Conclusions

The 2006 unemployment insurance reform led to drastic decreases of membership in insurance funds, reduced benefit levels, and stricter eligibility requirements – which in turn caused an increase in the proportion of unemployed individuals who did not receive any benefits. Our study has demonstrated that the reform had adverse effects on incidence rates of hospitalisation due to alcohol-related disorders among the unemployed, and especially for those who were Swedish-born (with Swedish-born or foreign-born parents), low-educated, or men – groups which are known to consume alcohol at relatively higher levels. This suggests that those who already engage in health-damaging behaviours are more vulnerable to external stressors such as the 2006 unemployment insurance reform. For future implementation of comprehensive reforms that decrease welfare state generosity and coverage, policy makers should consider accompanying interventions to address secondary consequences among vulnerable groups in society.

## Supplementary Information

**Additional file 1: Supplementary Figure S1.** Flowchart.

**Additional file 2: Supplementary Figure S2.** Unemployment benefit recipiency, stratified by ethnic background (ages 30-60, 2001–2012).

**Additional file 3: Supplementary Figure S3.** Unemployment benefit recipiency, stratified by educational level (ages 30-60, 2001–2012).

**Additional file 4: Supplementary Figure S4. **Unemployment benefit recipiency, stratified by sex (ages 30-60, 2001–2012).

**Additional file 5: Supplementary Table S1.** Results from the regression discontinuity models (ages 30-60, 2001–2012).

**Additional file 6: Supplementary Figure S5.** Regression discontinuity plots with incidence rates of alcohol-related disorders. Unemployed population, stratified by ethnic background (ages 30-60, 2001–2012). Regression lines show linear polynomials with cut offs at January 2007, January 2004, and January 2010.

**Additional file 7: Supplementary Figure S6.** Regression discontinuity plots with incidence rates of alcohol-related disorders. Unemployed population, stratified by educational level (ages 30-60, 2001–2012). Regression lines show linear polynomials with cut offs at January 2007, January 2004, and January 2010.

**Additional file 8: Supplementary Figure S7.** Regression discontinuity plots with incidence rates of alcohol-related disorders. Unemployed population, stratified by sex (ages 30-60, 2001–2012). Regression lines show linear polynomials with cut offs at January 2007, January 2004, and January 2010.

**Additional file 9: Supplementary Figure S8.** Regression discontinuity plots with incidence rates of alcohol-related disorders. Unemployed population, stratified by ethnic background (ages 30-60, 2005–2008).

**Additional file 10: Supplementary Figure S9.** Regression discontinuity plots with incidence rates of alcohol-related disorders. Unemployed population, stratified by educational level (ages 30-60, 2005–2008).

**Additional file 11: Supplementary Figure S10.** Regression discontinuity plots with incidence rates of alcohol-related disorders. Unemployed population, stratified by sex (ages 30-60, 2005–2008).

## Data Availability

The data that support the findings of this study are available from Statistics Sweden and the National Board of Health and Welfare but restrictions apply to the availability of these data, which were used under license for the current study, and so are not publicly available. Data are however available from the authors upon reasonable request and with permission of Statistics Sweden and the National Board of Health and Welfare.

## Abbreviations

ESMR: Evenly spaced mimicking-variance
ICD: International Classification of Diseases
LISA: Longitudinal Integration Database for Health Insurance and Labour Market Studies
RD: Regression discontinuity
RDD: Regression discontinuity design
RDiT: Regression discontinuity in time
STATIV: Longitudinal database for integration studies
