# Supplementary material for: The impact of an unemployment insurance reform on incidence rates of hospitalisation due to alcohol-related disorders: a quasi-experimental study of heterogeneous effects across ethnic background, educational level, employment status, and sex in Sweden
Source: BMC Public Health. 2022 Oct 3;22:1847. doi: 10.1186/s12889-022-14209-2 (PMC9531446; doi:10.1186/s12889-022-14209-2)
Supplement: Supplementary file 1 — Additional file 1: Supplementary Figure S1. Flowchart. [file 12889_2022_14209_MOESM1_ESM.docx]

**LISA database**: population age 30 -60

(2001 – 2012)

N=5,759,292

**Study population**

N=5,009,832

(42,370,094 person-years)

**Excluded (n=637,860)**

Died before entering the study: n=76,010

Immigrated after study period: n=140,042

Emigrated before study period: n=421,808

**Excluded (n=111,600)**

Missing ‘ethnic background’: n=5,642

Missing ‘education’: n=97,517

Uncertain unemployment status: n=8,441

Supplementary Figure S1. Flowchart.
