# Supplementary material for: The impact of an unemployment insurance reform on incidence rates of hospitalisation due to alcohol-related disorders: a quasi-experimental study of heterogeneous effects across ethnic background, educational level, employment status, and sex in Sweden
Source: BMC Public Health. 2022 Oct 3;22:1847. doi: 10.1186/s12889-022-14209-2 (PMC9531446; doi:10.1186/s12889-022-14209-2)
Supplement: Supplementary file 3 — Additional file 3: Supplementary Figure S3. Unemployment benefit recipiency, stratified by educational level (ages 30-60, 2001–2012). [file 12889_2022_14209_MOESM3_ESM.docx]

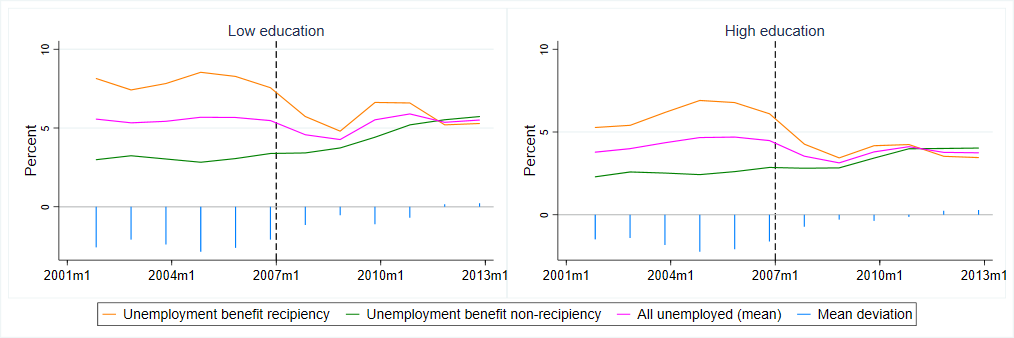


Supplementary Figure S3. Unemployment benefit recipiency, stratified by educational level (ages 30-60, 2001–2012).
