# Supplementary material for: The impact of an unemployment insurance reform on incidence rates of hospitalisation due to alcohol-related disorders: a quasi-experimental study of heterogeneous effects across ethnic background, educational level, employment status, and sex in Sweden
Source: BMC Public Health. 2022 Oct 3;22:1847. doi: 10.1186/s12889-022-14209-2 (PMC9531446; doi:10.1186/s12889-022-14209-2)
Supplement: Supplementary file 5 — Additional file 5: Supplementary Table S1. Results from the regression discontinuity models (ages 30-60, 2001–2012). [file 12889_2022_14209_MOESM5_ESM.docx]

Supplementary Table S1. Results from the regression discontinuity models (ages 30-60, 2001–2012).

|  | **Incidence rates of hospitalization due to alcohol-related disorders** | | | |
| --- | --- | --- | --- | --- |
|  | Estimate | P-value | Confidence interval | |
|  |  |  | Lower | Upper |
|  |  |  |  |  |
| **Total study population** | -0.49 | 0.000 | -0.58 | -0.40 |
| **Ethnic background** |  |  |  |  |
| Swedish-born with Swedish-born parents | -0.42 | 0.000 | -0.52 | -0.31 |
| Swedish-born with foreign-born parent(s) | 1.41 | 0.000 | 0.85 | 1.97 |
| European foreign-born | -2.39 | 0.000 | -2.95 | -1.82 |
| Non-European foreign-born | -0.62 | 0.000 | -0.77 | -0.48 |
| **Educational level** |  |  |  |  |
| Low education | -0.47 | 0.000 | -0.61 | -0.33 |
| High education | -0.43 | 0.000 | -0.47 | -0.38 |
| **Employment status** |  |  |  |  |
| Employed | -0.56 | 0.000 | -0.66 | -0.46 |
| Unemployed | 1.04 | 0.000 | 0.71 | 1.38 |
| **Sex** |  |  |  |  |
| Men | -0.77 | 0.000 | -0.94 | -0.60 |
| Women | -0.21 | 0.000 | -0.25 | -0.16 |
|  |  |  |  |  |
| **Unemployed population** |  |  |  |  |
| **Ethnic background** |  |  |  |  |
| Swedish-born with Swedish-born parents | 2.25 | 0.000 | 1.63 | 2.87 |
| Swedish-born with foreign-born parent(s) | 7.41 | 0.000 | 5.12 | 9.69 |
| European foreign-born | 3.83 | 0.000 | 2.15 | 5.52 |
| Non-European foreign-born | -2.05 | 0.000 | -2.50 | -1.61 |
| **Educational level** |  |  |  |  |
| Low education | 2.50 | 0.000 | 1.82 | 3.19 |
| High education | -1.52 | 0.000 | -2.02 | -1.01 |
| **Sex** |  |  |  |  |
| Men | 2.77 | 0.000 | 1.96 | 3.57 |
| Women | 0.51 | 0.000 | 0.23 | 0.78 |
