# Supplementary material for: The impact of an unemployment insurance reform on incidence rates of hospitalisation due to alcohol-related disorders: a quasi-experimental study of heterogeneous effects across ethnic background, educational level, employment status, and sex in Sweden
Source: BMC Public Health. 2022 Oct 3;22:1847. doi: 10.1186/s12889-022-14209-2 (PMC9531446; doi:10.1186/s12889-022-14209-2)
Supplement: Supplementary file 11 — Additional file 11: Supplementary Figure S10. Regression discontinuity plots with incidence rates of alcohol-related disorders. Unemployed population, stratified by sex (ages 30-60, 2005–2008). [file 12889_2022_14209_MOESM11_ESM.docx]

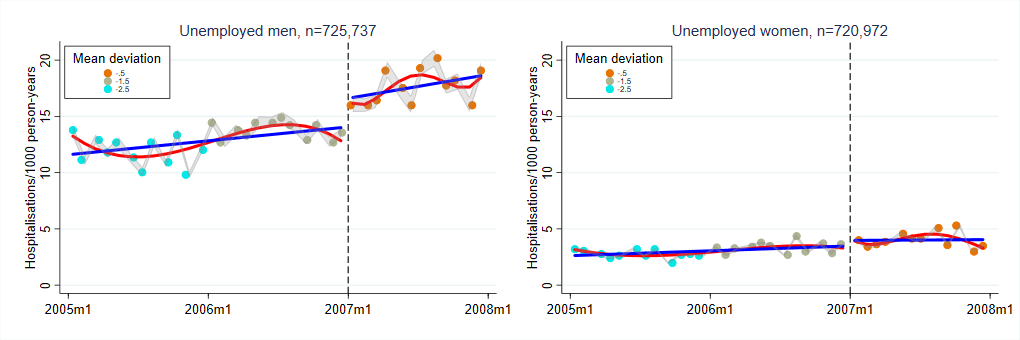


Supplementary Figure S10. Regression discontinuity plots with incidence rates of alcohol-related disorders. Unemployed population, stratified by sex (ages 30-60, 2005–2008).
